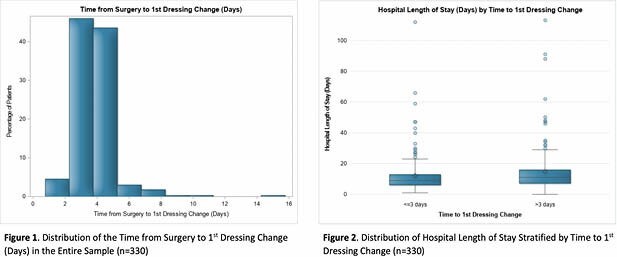# 51 Relationship of Early Dressing Change, Ambulation, and Early Discharge in Lower Extremity Grafted Burn Wounds

**DOI:** 10.1093/jbcr/irad045.025

**Published:** 2023-05-15

**Authors:** Sandor Toledo, Sigrid Blome-Eberwein, Cesar Barros De Souza, Harry Shi, Miname Watanabe, Morgan Anderson

**Affiliations:** Lehigh Valley Health Network, Allentown, Pennsylvania; Lehigh Valley Health Network, Allentown, Pennsylvania; Lehigh Valley Health Network, Allentown, Pennsylvania; Lehigh Valley Health Network, Allentown, Pennsylvania; Lehigh Valley Health Network, Allentown, Pennsylvania; Lehigh Valley Health Network, Allentown, Pennsylvania; Lehigh Valley Health Network, Allentown, Pennsylvania; Lehigh Valley Health Network, Allentown, Pennsylvania; Lehigh Valley Health Network, Allentown, Pennsylvania; Lehigh Valley Health Network, Allentown, Pennsylvania; Lehigh Valley Health Network, Allentown, Pennsylvania; Lehigh Valley Health Network, Allentown, Pennsylvania; Lehigh Valley Health Network, Allentown, Pennsylvania; Lehigh Valley Health Network, Allentown, Pennsylvania; Lehigh Valley Health Network, Allentown, Pennsylvania; Lehigh Valley Health Network, Allentown, Pennsylvania; Lehigh Valley Health Network, Allentown, Pennsylvania; Lehigh Valley Health Network, Allentown, Pennsylvania; Lehigh Valley Health Network, Allentown, Pennsylvania; Lehigh Valley Health Network, Allentown, Pennsylvania; Lehigh Valley Health Network, Allentown, Pennsylvania; Lehigh Valley Health Network, Allentown, Pennsylvania; Lehigh Valley Health Network, Allentown, Pennsylvania; Lehigh Valley Health Network, Allentown, Pennsylvania; Lehigh Valley Health Network, Allentown, Pennsylvania; Lehigh Valley Health Network, Allentown, Pennsylvania; Lehigh Valley Health Network, Allentown, Pennsylvania; Lehigh Valley Health Network, Allentown, Pennsylvania; Lehigh Valley Health Network, Allentown, Pennsylvania; Lehigh Valley Health Network, Allentown, Pennsylvania; Lehigh Valley Health Network, Allentown, Pennsylvania; Lehigh Valley Health Network, Allentown, Pennsylvania; Lehigh Valley Health Network, Allentown, Pennsylvania; Lehigh Valley Health Network, Allentown, Pennsylvania; Lehigh Valley Health Network, Allentown, Pennsylvania; Lehigh Valley Health Network, Allentown, Pennsylvania

## Abstract

**Introduction:**

Split-thickness skin graft (STSG) healing follows the imbibition, inoculation, and revascularization healing model. The stage of imbibition takes approximately 48-72 hours and requires direct contact with the wound for success. Prior to revascularization the graft is at increased risk for damage from shear stress. Many of the protocols in place are based on pathophysiology of wound healing and few published data exist on the timing and logistics of mobilization after lower extremity STSG for burns. There is discordance in the Burn community as to when it is safe to start ambulation with patients after lower extremity skin grafting. The timing of the first dressing change may accelerate or delay ambulation and increase time to hospital discharge.

**Methods:**

Retrospective chart review of burn patients regardless of age, admitted to burn service with grafted burns to lower extremities, whether they had other concomitant burns with/without grafting to determine if earlier first postoperative dressing changes (≤ 3 days) to grafted lower extremity burns lead to earlier ambulation and shorter hospital stays. Secondary endpoints will be evaluation of the ideal time to change the dressing after initial STSG grafting of the lower extremity without increasing graft failure rates.

**Results:**

The groups were even in time from surgery to 1^st ^dressing change (1^st^ DC) (167 had a 1^st^ DC in ≤3 days post-op, and 163 had a 1^st^ DC >3 days post-op). Demographics and medical history were nearly identical between the two groups, indicating there may be no association between the demographics or medical history collected and earlier dressing change. Median time from surgery to the 1^st^ DC was 3 days (IQR: 3-5) in the entire sample, and from surgery to staple removal was 5 days (IQR: 4-6). The median length of hospital stay in the entire sample was 10 days, and this slightly differed between groups: those with a 1^st^ DC of ≤3 days had a shorter hospital stay (9 days [IQR: 6-13]) than those with a 1^st^ DC of >3 days (11 days [IQR: 7-16]), this is exemplified in Figure 2. 80.3% of patients were discharged home in the sample, with a greater proportion of patients discharged to a facility when they had a 1^st^ DC >3 days post-op (24.5%) than if they had a 1^st^ DC ≤3 days post-op (15%). A greater proportion of patients were discharged walking (47.6% vs 32.7%) when they had an earlier 1^st^ DC than those who did not. Graft failure was seen in 6 patients with 5 of them needing re-STSG. Four due to graft loss and two due to cellulitis.

**Conclusions:**

Patients who had 1^st^ DC ≤ 3 days after surgery saw an earlier hospital discharge by 2 days without risk of graft loss due to hematoma or seroma regardless of comorbidities.

**Applicability of Research to Practice:**

The data gathered will be used to establish a clinical based approach to lower extremity dressing changes. We hope to delineate a timeline based on tailored patient factors that can be used as guideline to safely expedite ambulation and minimize hospital stays without increasing graft failure rates.